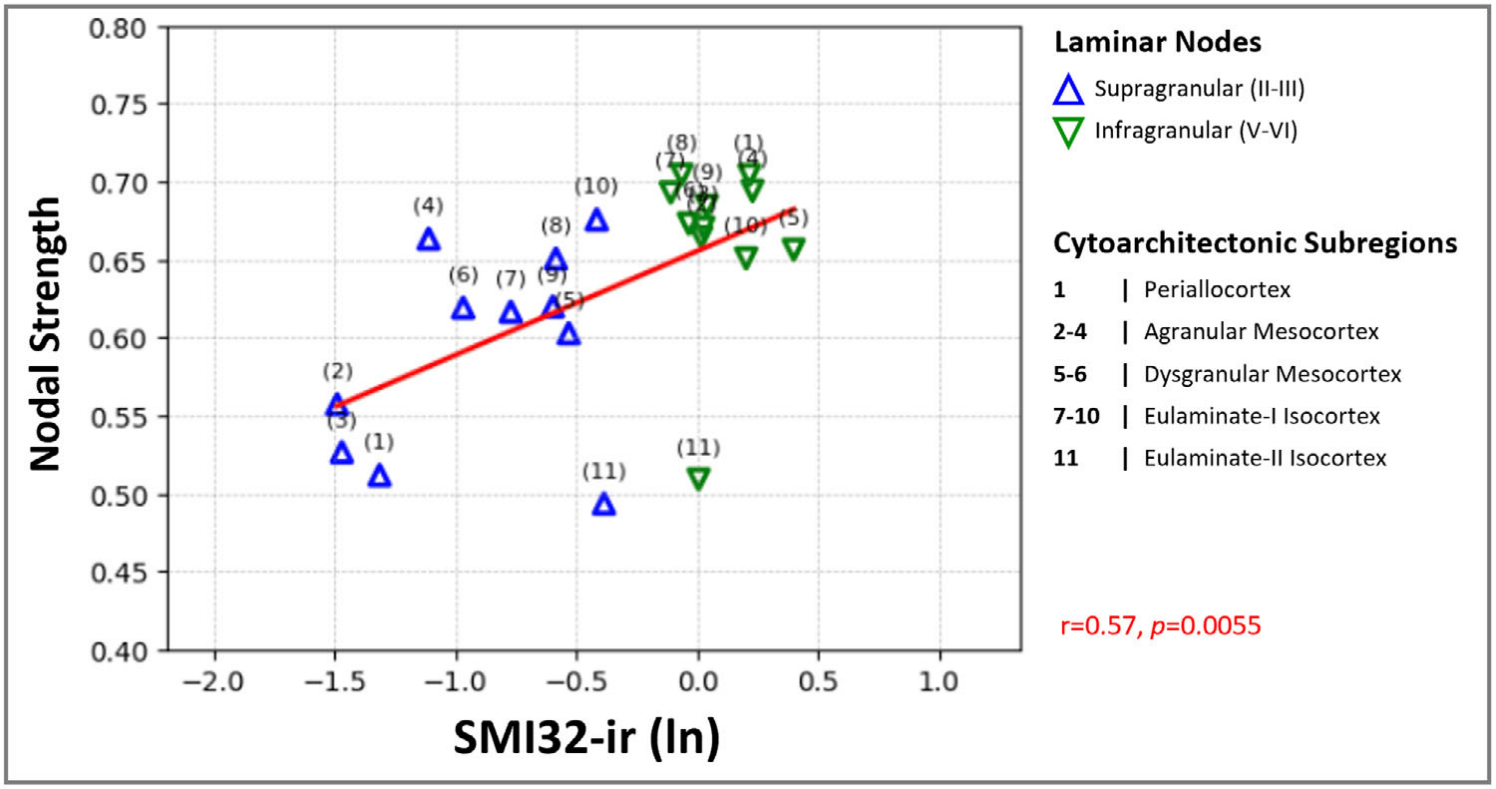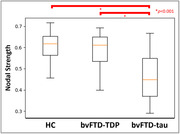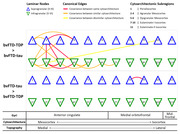# Distinct laminar networks of neuronal covariance in behavioral variant frontotemporal dementia with either tau or TDP‐43 pathology

**DOI:** 10.1002/alz.095246

**Published:** 2025-01-09

**Authors:** Daniel T Ohm, Hyung Seok Roh, Min Chen, Sanaz Arezoumandan, Jeffrey S Phillips, Katheryn A Q Cousins, David A Wolk, Eddie B Lee, Corey T McMillan, James C. Gee, David J Irwin

**Affiliations:** ^1^ Penn Frontotemporal Degeneration Center, Department of Neurology, Perelman School of Medicine, University of Pennsylvania, Philadelphia, PA USA; ^2^ Digital Neuropathology Laboratory, University of Pennsylvania, Philadelphia, PA USA; ^3^ Penn Image Computing and Science Laboratory, Philadelphia, PA USA; ^4^ Penn Memory Center, University of Pennsylvania, Philadelphia, PA USA; ^5^ Penn Alzheimer’s Disease Research Center, University of Pennsylvania, Philadelphia, PA USA; ^6^ Center for Neurodegenerative Disease Research, Perelman School of Medicine, University of Pennsylvania, Philadelphia, PA USA; ^7^ Translational Neuropathology Research Laboratory, Perelman School of Medicine, University of Pennsylvania, Philadelphia, PA USA

## Abstract

**Background:**

Structural covariance analyses have identified macrostructural/morphological alterations to MRI‐based networks in behavioral variant frontotemporal dementia (bvFTD), but microstructural/neuronal alterations to histology‐based networks remain unexplored. We previously found greater neurodegeneration in layers and regions enriched for pyramidal neurons in bvFTD with tau (bvFTD‐tau) compared to TDP‐43 (bvFTD‐TDP) pathology. Therefore, we hypothesized laminar networks of empirically connected pyramidal neurons are weaker in bvFTD‐tau versus bvFTD‐TDP.

**Method:**

In clinically similar bvFTD‐tau (n = 27) and bvFTD‐TDP (n = 47) and age‐matched healthy controls (HC; n = 32), we digitally quantified SMI32‐immunoreactive (ir) pyramidal neurons in supragranular (II‐III) and infragranular (V‐VI) layers of up to 11 frontal regions spanning anterior cingulate, paracingulate, orbitofrontal, and mid‐frontal gyri and comprising a diverse range of mesocortical‐to‐isocortical cytoarchitecture, including periallocortex, agranular mesocortex, dysgranular mesocortex, eulaminate‐I isocortex, eulaminate‐II isocortex. Using graph theory, we constructed group‐level covariance networks composed of 22 *nodes* (laminar subregions) and *weighted edges* (Pearson correlations of SMI32‐ir between nodes) representing 231 possible laminar connections. We analyzed an empirically‐derived subnetwork (n = 159 edges) comprising three types of laminar connections: 1) within same cytoarchitecture, 2) between similar cytoarchitecture (neighboring gyri), 3) between dissimilar cytoarchitecture (distant gyri). Pearson correlations compared SMI32‐ir to *nodal strength* (NS), a metric of network integrity where summations of weighted edges are divided by total possible edges per node. One‐way ANOVA with Tukey’s Honest Significant Difference post‐hoc test compared NS between groups and Fisher Z‐transformation tested for significantly weaker edges between groups.

**Result:**

Analysis of all possible edges found lower SMI32‐ir associated with lower NS (r = 0.57, p = 0.0055), consistent with previous antemortem network studies suggesting neurodegeneration reduces network integrity (Fig. 1). In canonical subnetworks, groups showed different NS (F[2,63] = 19.45, p<0.001), including lower NS in bvFTD‐tau versus bvFTD‐TDP (beta = ‐0.13, p<0.001) and HC (beta = ‐0.15, p<0.001), but similar NS between bvFTD‐TDP and HC (p = 0.686), (Fig. 2). Canonical edge comparisons between bvFTD‐TDP and bvFTD‐tau found one weaker edge between neighboring supragranular isocortices in bvFTD‐TDP, and 18 weaker edges between predominantly similar/neighboring mesocortices and select dissimilar/distant isocortices in bvFTD‐tau (Fig. 3).

**Conclusion:**

Among clinically similar bvFTD patients, pyramidal neurodegeneration produces weaker laminar networks in bvFTD‐tau versus bvFTD‐TDP, suggesting tauopathies may preferentially weaken/degenerate corticocortical microcircuits linking local and distant frontal cytoarchitecture.